# Characterizing pearls structures using X-ray phase-contrast and neutron imaging: a pilot study

**DOI:** 10.1038/s41598-018-30545-z

**Published:** 2018-08-14

**Authors:** D. Micieli, D. Di Martino, M. Musa, L. Gori, A. Kaestner, A. Bravin, A. Mittone, R. Navone, G. Gorini

**Affiliations:** 1 Dip. di Fisica “G. Occhialini”, Università degli Studi Milano-Bicocca, Piazza della Scienza 3, 20126 Milano Italy; 2 Dip. di Fisica, Università della Calabria, via Pietro Bucci, 87036 Arcavacata di Rende (Cosenza), Italy; 3 GECI - Gemological Education and Certification Institute, Via delle Asole 2, 20123 Milano Italy; 4 Laboratory for Neutron Scattering and Imaging, Paul Scherrer Institute, 5232 Villigen, Switzerland; 5 European Synchrotron Radiation Facility, 38043 Grenoble, France; 6 R.A.G. - Laboratorio di analisi e consulenze gemmologiche, Corso San Maurizio 52, 10124 Torino Italy

## Abstract

Some cultured and natural pearls can be reliably distinguished by visual inspection and by the use of lens and microscope. However, assessing the origin of the pearls could be not straightforward since many different production techniques can now be found in the pearl market, for example in salt or freshwater environments, with or without a rigid nucleus. This wide range of products requires the use of new effective scientific techniques. Indeed, X-ray radiography has been used by gemologists since last century as the only safe and non-destructive way to visually inspect the interior of a pearl, and recently, also X-ray computed micro-tomography was used to better visualize the inner parts of the gems. In this study we analyzed samples of natural and cultured pearls by means of two non-destructive techniques: the X-ray Phase-Contrast Imaging (PCI) and the Neutron Imaging (NI). PCI and NI results will be combined for the first time, to better visualize the pearls internal morphology, thus giving relevant indications on the pearl formation process.

## Introduction

Natural pearls were considered precious and rare gems for many centuries. There is an important price gap between the two kinds of pearls: cultured pearls may cost a few hundreds of dollars while the natural pearls can be sold for millions of dollars. As the culturing techniques have improved, the distinction between natural and cultured pearls has become a challenging task and still an open question for gemologists^1–4^. Apart from obvious cases of internal structures evidenced clearly by using the traditional methods, there are cases in which better resolution and sensitivity are needed to highlight the subtle characteristics necessary for pearl identification.

The biological mechanisms of molluscs responsible for pearl creation are the same of shell secretion. All pearls are very complex biogenetic structures^5^, consisting of inorganic matrix-mediated mineralization, mainly of calcium carbonate in calcite and aragonite phases, performed by living tissues. The calcifying matrix is a mixture of proteins, glycoproteins and polysaccharides that precisely self-assemble and control the CaCO_3_ polymorph, the size, the shapes of the crystals and their orientation^6^. The complexity of these structures is proved also by the fact that not all the biochemical reactions involved during the pearl genesis are understood yet.

For decades, the analysis of pearls was mainly based on the X-ray radiography^7,8^ that allows to visualize slight variations in X-ray absorption. However, this technique is often inefficient to reveal small intrinsic structure variations of pearls. Only recently, the X-ray computed micro-tomography was used to better reconstruct the inner parts of the gems^9–11^. Other techniques involving X-ray laboratory sources and based on the so called phase contrast mechanism were tested^12,13^ and compared with neutron imaging techniques^14,15^. Recently, an energy resolved neutron imaging study on pearls was carried out to analyse the phase composition^16^. Yet, the newest pearl culturing techniques may require a combination of multiple test methods.

In this study, two non-destructive techniques, the X-ray Phase-Contrast Imaging (PCI) - performed at ID17 biomedical beamline, European Synchrotron Radiation Facility (ESRF) - and the Neutron Imaging (NI) - performed at ICON beamline, Paul Scherrer Institute (PSI), were applied to the study of very complex structures like the pearl specimens. Synchrotron Radiation Phase-Contrast Imaging (SR-PCI) combined with NI allowed visualizing the inner morphology of the pearl in great detail, giving new insights into the growth process of pearls.

PCI basically takes advantage of the phase shift that occurs when an X-ray beam passes through an object^17^. This class of techniques has been shown to lead to a superior image contrast compared to the conventional X-ray attenuation-based imaging, especially for low atomic number samples, and therefore including biological samples. On the other hand, neutrons are strongly attenuated by hydrogen and easily transmitted through metals; as a consequence NI gives complementary results respect to X-ray imaging. In this regard, neutrons could be a suitable probe to observe the various kind of pearl structures and differentiate organic tissues from inorganic carbonate phases and, possibly, also disclose the origin of pearls in uncertain cases.

## Material and Methods

### Samples

**Figure 1:**
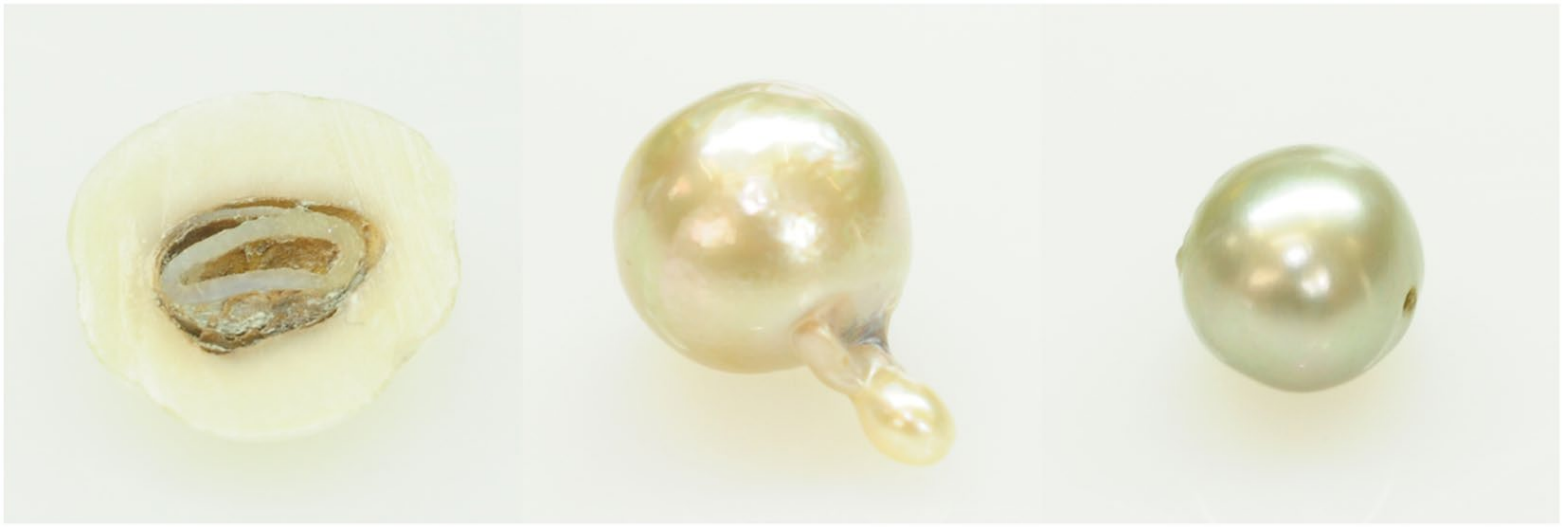
(From left to right) the natural pearl NP-22, the bead saltwater pearl CP-17 and the non-bead freshwater cultured pearl CP-21.

In this work we chose three different pearls as a case study: a natural pearl, a bead cultured pearl and a non-bead cultured pearl. Images of the samples are displayed in Fig. 1 and the most important gemological features are described in Table 1.

**Table 1:** Main gemological characteristics of the studied pearl samples.

Sample n.	NP-22	CP-17	CP-21
Species and variety	Natural Pearl	Bead Cultured Pearl	Non-Bead Cultured Pearl
Environmental	Saltwater	Saltwater	Freshwater
Shape	Semi-Baroque circled	Baroque	Baroque
Measurements	7.84 × 8.63 × 4.06 mm	10.30–10.34 × 15.37 mm	7.95–8.13 × 9.45 mm
Weight	1.78 ct	8.23 ct	4.40 ct
Drilling	sewed	none	Drilled
Body colour	Cream	Cream	Grey
Overtone	—	Green - Rosee	Green
Luster	Good	Good	Fair
Surface quality	Heavily spotted	Moderately spotted	Lightly Spotted

NP-22 is a vintage pearl with a negligible commercial value from a private collection and it has been sewed for didactical purposes. This pearl in fact presents an odd inner structure different from the classical “onion-like” morphology, indeed aspects of natural pearl formation remain a mystery in many cases^18,19^. The sewing process could have partially modified the core appearance, but this is a secondary aspect for our work purpose. Therefore, sewed NP-22 represents a very suitable sample to test the imaging techniques due to its well visible complex structures.

On the other hand, CP-21 is a classic example of non-bead freshwater cultured pearl: this type of gemological material is very cheap and common on the market, but in some cases the classic gemological tests are not enough to distinguish between natural and this type of cultured pearls.

Finally, CP-17 presents typical bead and therefore is easy to identify it as cultured pearl. However, it has been chosen for our study because the structure is characterized by the presence of a small additional “pearl”, generated by detachment of mantle tissue portion during the starting pearl generation phases. These events sometimes are considered an incidental precursor step of “keshi” generation pearls^20^. Keshi (in Japanese literally “poppy seed”) is the trade name for cultured pearl which had formed accidentally in marine bivalve mollusks used for bead cultured pearl or in cultured pearls which were produced by inserting a small piece of mantle tissue^21^.

### Neutron tomography

Neutron Tomography (NT) is a non-destructive technique that provides the three-dimensional map of the neutron attenuation coefficient within a sample^22,23^. It consists in collecting a set of transmission radiographs at different angular views of the sample by rotating it, generally with small uniform increments over 180 or 360 degrees. The three-dimensional map of the attenuation coefficient is computed from a set of radiographs by means of mathematical reconstruction algorithms^24,25^. NI has unique capabilities with respect to X-ray imaging, such as the high-sensitivity to hydrogenous materials, the penetration of thick metals and the differentiation of isotopes of the same element or neighbour elements in the periodic table. This means that the combined use of Neutron Imaging (NI) and X-ray Imaging gives complementary information^26,27^.

### Data acquisition at ICON beamline

The NI investigation was carried out at the beamline for Imaging with COld Neutrons (ICON)^28^, at the Swiss spallation neutron source (SINQ), Paul Scherrer Institute (PSI), Switzerland. The pearls were placed in a tube-like sample holder and aluminium foil was used as spacing between the pearls and to fix a good positioning that prevents accidental motions during the scan. The neutron beam generated by SINQ reaches the sample, placed at L = 6864 mm from the source, with a flux of 1.3 10^7^ $\,\frac{{\rm{neutrons}}}{c{m}^{2}\,s\,}$ for the selected aperture of D = 20 mm that defines a L/D ratio of 343. The detection system consisted of a 16-bit CCD camera with 2048 × 2048 pixels (ANDOR DV436) coupled to a Gd-based scintillator with thickness of 20 $\mu m$. The resolution achieved for this setup is 27.8 $\mu m$ with a Field Of View (FOV) of 27 × 27 mm^2,29^. Each tomographic scan was performed by collecting a set of 626 radiographs in the angular range [0°, 360°], with an exposure time of 90 s for each projection and an overall scan time of approximately 20 hours. Open beam and dark images were taken as well in order to fulfil the flat-field correction.

The data pre-processing and reconstruction were performed by means of MuhRec^30^, a software developed to support the need of users at neutron imaging beamlines. Some pre-processing steps are necessary for the right evaluation of the reconstructed images: the normalization of the projection respect to dark and open beam images, the suppression of spots caused by gamma rays hitting the CCD sensor, the computation of the rotation axis position and the ring removal by means of a filter based on combined wavelet and Fourier analysis^31^. The tomographic reconstruction was performed by means of the Filtered Back Projection (FBP) algorithm for a parallel beam geometry^24^. However, the reconstructed images are affected by noise that must be suppressed for a straightforward segmentation and analysis. Hence, an Inverse Scale Space filter^32^ was applied to reconstructed images by using the image processing software KipTool^33^.

### X-ray Phase Contrast Imaging

PCI relies on the beam phase shift occurring when X-rays pass through a sample and resulting in intensity variations recorded by a detector. Phase sensitive imaging allows obtaining higher image contrast at a lower radiation dose with respect to conventional attenuation-based imaging. Several X-ray PCI techniques have been developed in the last decades and an exhaustive description can be found in^17,34^.Among the possible PCI techniques, the one used in this work is called propagation-based imaging (PBI)^35,36^. A highly spatially coherent and quasi-monochromatic X-ray beam irradiates the object, placed at a suitable distance from the detection system, and, thanks to the Fresnel diffraction, the interfaces between the different materials are strongly highlighted by the generated interference fringes.

### Data acquisition at ID17

The X-ray PCI investigation was carried out at the ID17 beamline of the ESRF in Grenoble (France)^37^. A 21-pole wiggler was used together with a double Si Laue crystal monochromator. The obtained X-ray beam is quasi-monochromatic (ΔE/E ∼10^−4^) and quasi-parallel, presenting a divergence <1 mrad, horizontally, and <0.1 mrad, vertically. The imaging detector was a PCO edge 5.5, a 16 bit sCMOS camera with 2560 × 2160 pixels, coupled with a 2x optics and a 350 $\mu m$ thick YAG scintillator, resulting in a final pixel size of 3.1 $\mu$m^38^. The samples were placed at the distance of 2.3 m from the detection system and imaged with an X-ray energy of 50 keV. Each tomographic scan was performed using the half-acquisition CT mode that allows almost to double the width of the field of view (FOV). Hence, sets of 2500 angular projections were collected in the range [0°, 360°) in the case of the cultured pearls, while 5000 radiographs were acquired for the scan of the natural pearl. Due to the vertically limited FOV and beam size, in order to scan the entire cultured pearls that are bigger than the natural pearl, two tomographic measurements were acquired at two different vertical positions.

The data processing and reconstruction were performed by using SYRMEP Tomo Project (STP)^39^, an open-source software tool conceived for post-beamtime, allowing the users to design custom reconstruction workflows of archived data. In the pre-processing stage, the data were normalized by using the dark and flat-field images and processed by a ring suppression filter^31^. Prior the CT reconstruction, each normalized projection must be processed to extract the phase information by means of a phase retrieval procedure^40^. For this purpose the Paganin’s algorithm^41^, implemented in STP, has been used. Finally, also in this case, the tomographic reconstruction was performed by means of the FBP algorithm for parallel beam geometry.

## Results and Discussion

The 3D rendering and the registration of the X-ray data respect to neutron data were performed using the Avizo 8 software^42^. In this section we present for each pearl the results and a detailed description of the visible structures disclosed by both techniques. We recall that each NI image represents a two-dimensional map of the attenuation coefficient, so brighter pixels represent a higher neutron attenuation. The X-ray PC images depict the refractive index decrement distribution, which is proportional to the electron density. Hence, in this case, brighter zones correspond to a higher electron density.

### Pearl NP-22

**Figure 2:**
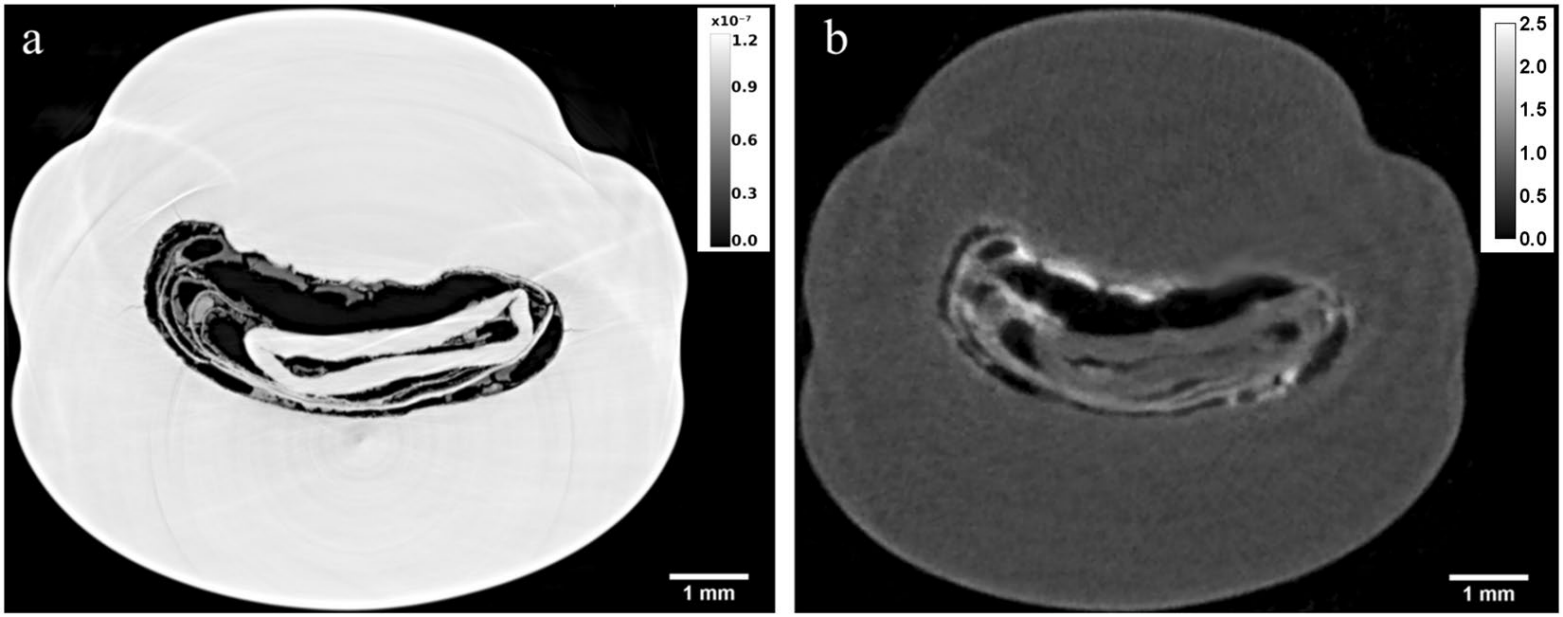
X-ray PC (**a**) and neutron (**b**) reconstructed image corresponding to the same slice of the natural pearl NP-22. Aragonite phases figure as white and dark grey zones in the X-ray and neutron images, respectively. Organic matter is recognized as dark grey and white zones in the X-ray and neutron images, respectively. In the neutron image an elevate concentration of organic matter has been identified, visible as white zones due to hydrogen presence. On the other hand, the X-Ray PC image shows with more details the concentric growth arcs and the layers of organic matter. Grey scale refers to refractive index decrement for PCI (**a**) and to linear attenuation coefficient (cm^−1^) for NI (**b**).

**Figure 3:**
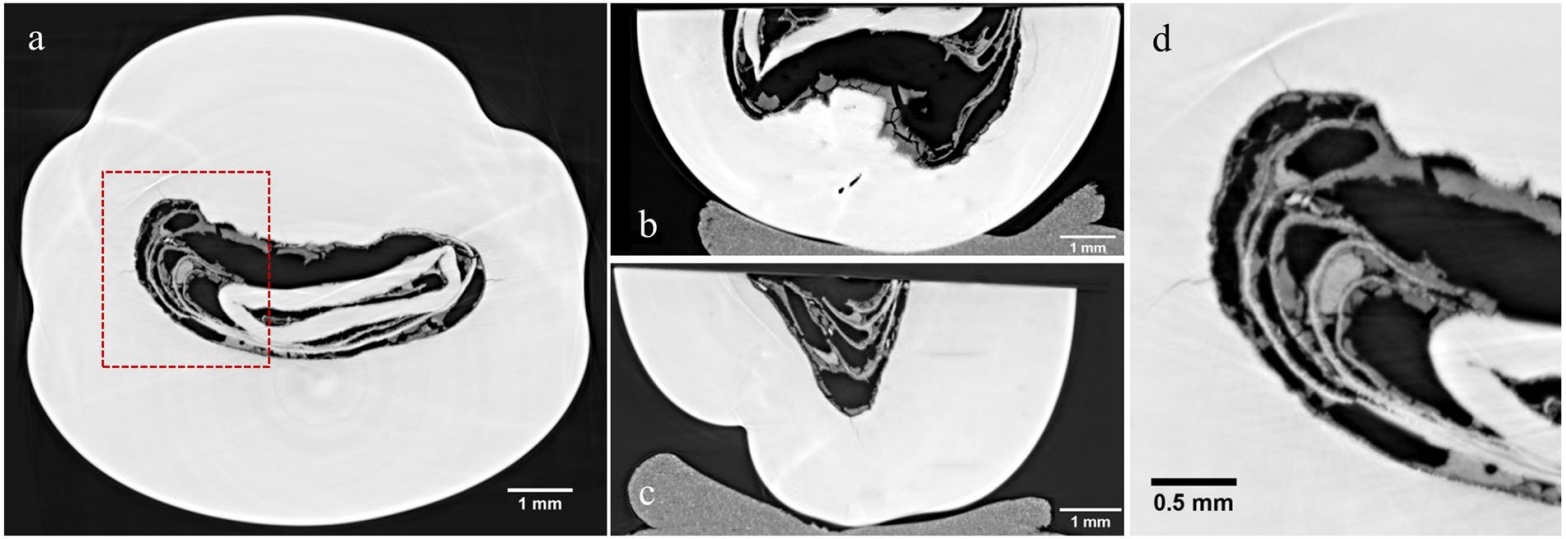
Three orthogonal X-ray PC slices (**a**–**c**) and a detail (**d**) of the slice represented in (**a**). Small fissures and pearl structures are clearly visible, as well as (in light grey) a carborundum contamination due to the sewing.

**Figure 4:**
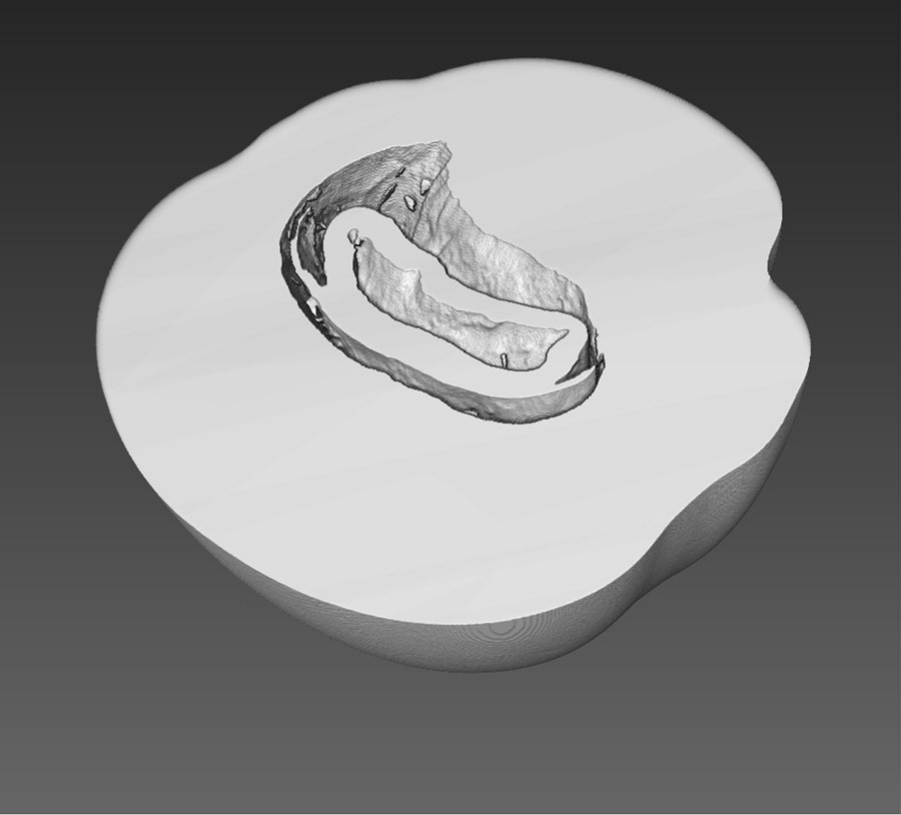
Volume rendering of the aragonite structure of the natural pearl NP-22 obtained from X-ray PC data.

Figure 2a,b show an X-ray PC image and a neutron image corresponding to the same slice of the natural pearl NP-22. Both reconstructed images reveal the complex inner structure composed of calcium carbonate and organic matter organized in several layers. The aragonite phase, identified by additional micro-Raman spectroscopy analyses performed on the sewed pearl surface^43^, are clearly visible in X-ray PC image as white zones, conversely in neutron images they appear as dark grey regions. The organic matter figures as white stain in neutron images and as dark grey in X-ray PC images. However, the X-ray PC offers the best results in terms of resolution and detects some details that cannot be disclosed by NT. In fact, Fig. 3a–c show three orthogonal X-ray PC slices of the natural pearl where a third phase is visible as light grey, attributable to a carborundum contamination due to the sewing. The internal portion of pearl shows concentric growth arcs, in conjunction with several layers of organic matter. The resolution of few microns gives the possibility to visualize small fissures in the inner part of the pearl (Fig. 3d), which are usually due to the dehydration or the ageing of the pearl. Both tomographic reconstructions allow to identify not just the nacre composed of aragonite, but also aragonite structures in the core of the pearl that cannot be disclosed with neutron or X-ray radiography. These parts of the natural pearl NP-22 were segmented by choosing the proper grey values thresholds for the calcium carbonate phase, after the application of a denoising filter. The 3D rendering of the aragonite phase starting from X-ray PC data is shown in Fig. 4.

### Pearl CP-17

**Figure 5:**
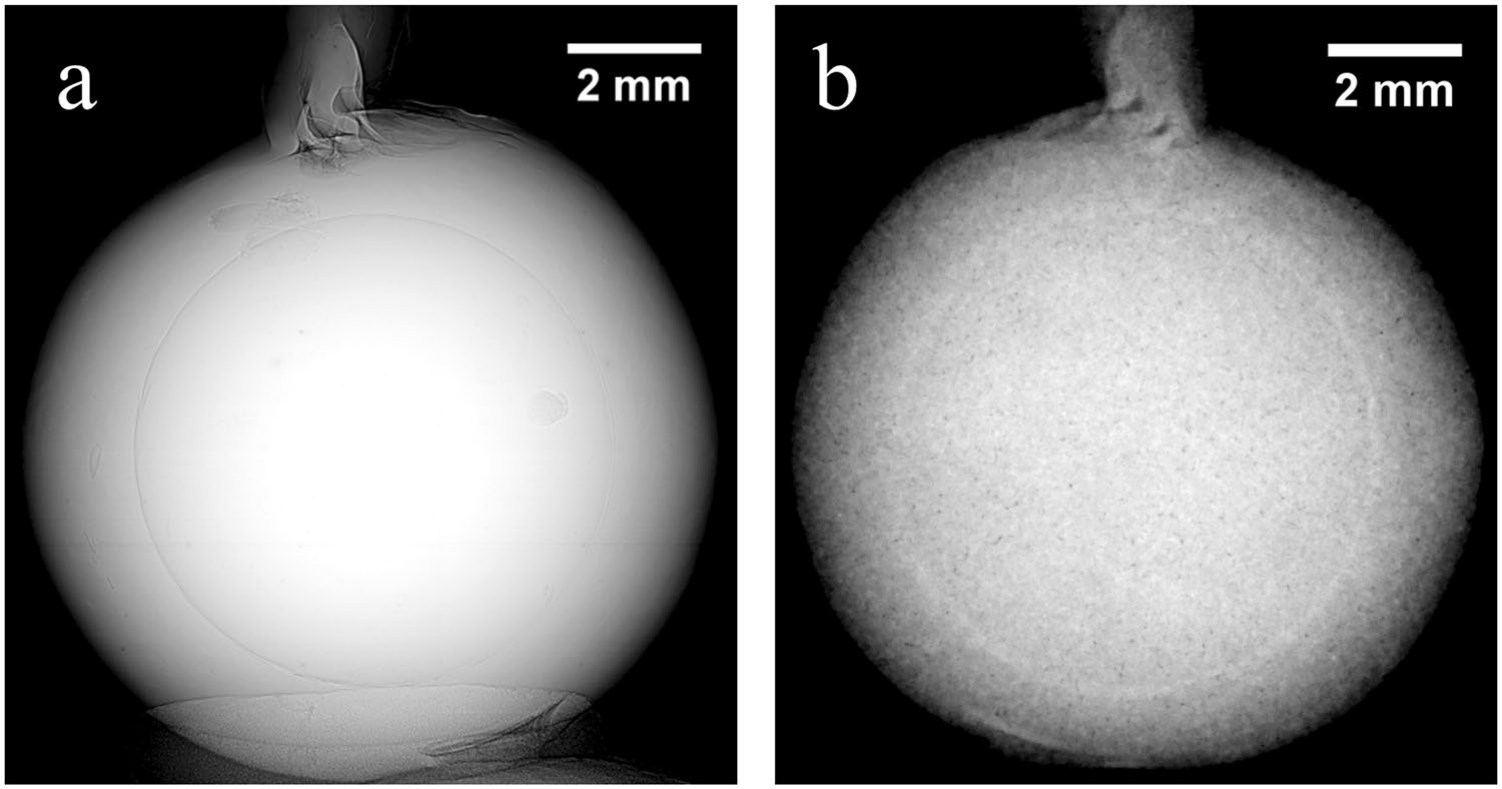
X-ray PC (**a**) and neutron (**b**) radiographs of the beaded saltwater pearl CP −17. A ring, due to contact surface between nacre and bead, is recognized in both radiographs.

**Figure 6:**
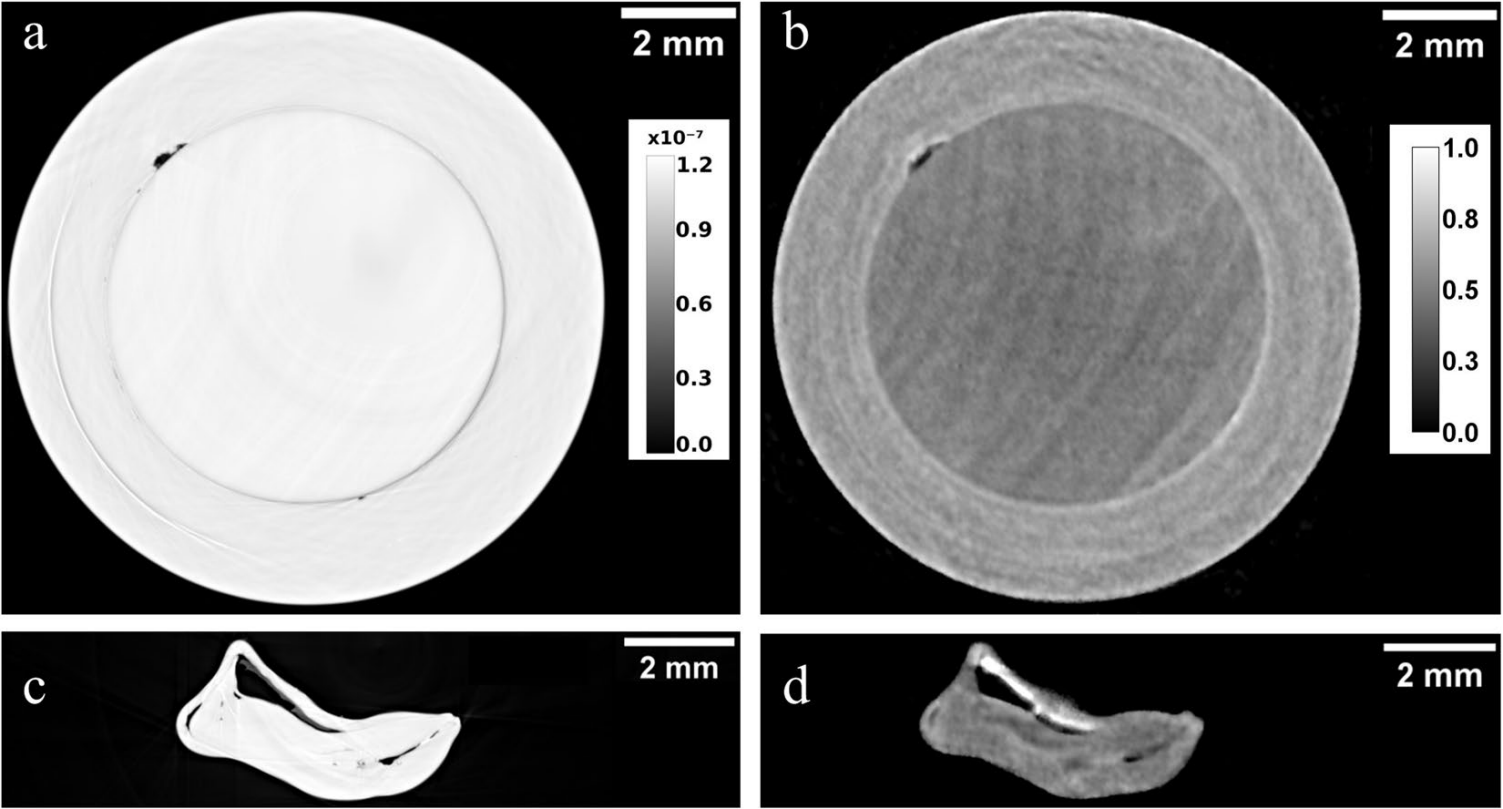
X-ray PC (**a**) and neutron (**b**) CT reconstructed images corresponding to a slice placed in the middle of the main part of the pearl CP-17. X-ray PC (**c**) and neutron (**d**) reconstructed images corresponding to a slice cutting the small attached pearl of CP-17. Organic tissue within the small attached pearl is recognized as grey zones in the neutron image (**d**) and white in the X-ray PC image (**c**). Grey scale refers to refractive index decrement for PCI (**a**) and to linear attenuation coefficient (cm^−1^) for NI (**b**).

**Figure 7:**
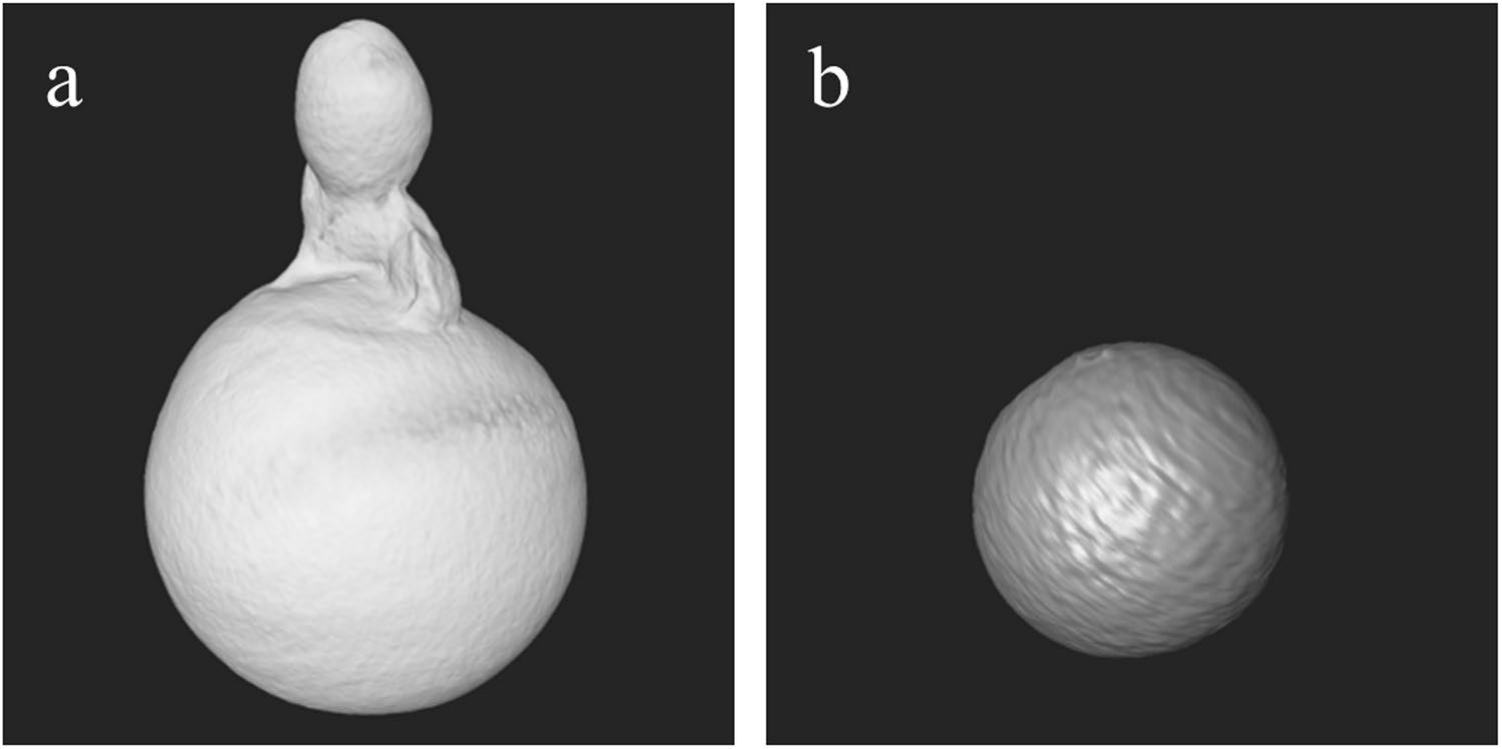
Volume rendering of the whole pearl CP-17 (**a**) and the inner bead (**b**) starting from neutron data.

The identification of the beaded saltwater pearl CP-17 is straightforwardly performed with X-ray PC and neutron radiography. In fact, an X-ray and a neutron radiograph of the pearl CP-17 shown in Fig. 5a,b reveal a ring in both images, as a result of the contact surface between nacre and bead. The tomographic reconstruction gives additional information about the distribution of organic tissues, voids and calcium carbonate phases. Figure 6a,b show the X-ray and neutron image of a slice placed in the middle of the main part of the pearl, while Fig. 6c,d display the images of a slice that crosses the small additional “pearl”. The bead can be recognised again in both reconstructed images (Fig. 6a,b) and we observe in the neutron image (Fig. 6b) that the neutron attenuation of the bead is lower than the attenuation of the nacre (better evidenced by the neutron technique, probably for the different organic component). Note also in Fig. 6b the parallel layers visible in the bead, characteristic of shell structure and organized in different orientation respect those observed in nacre of CP-17; in fact the nucleus, commonly made of freshwater mother-of-pearl shell, consists in parallel layers of aragonite platelets^21^. Hence, in this case the segmentation of the pearl was performed with neutron data and the volume rendering of the whole pearl and its bead are shown in Fig. 7a,b, respectively. However, the X-ray PC tomography reveals small voids and structures (Fig. 6a–c), related to the mantle tissue who originated the small additional pearl, not seen by the NT, due its lower spatial resolution. Both tomographic reconstructions allow identifying organic tissue within the small attached pearl that appears as grey in the neutron images and white in the X-ray PC images (Fig. 6c,d).

### Pearl CP-21

**Figure 8:**
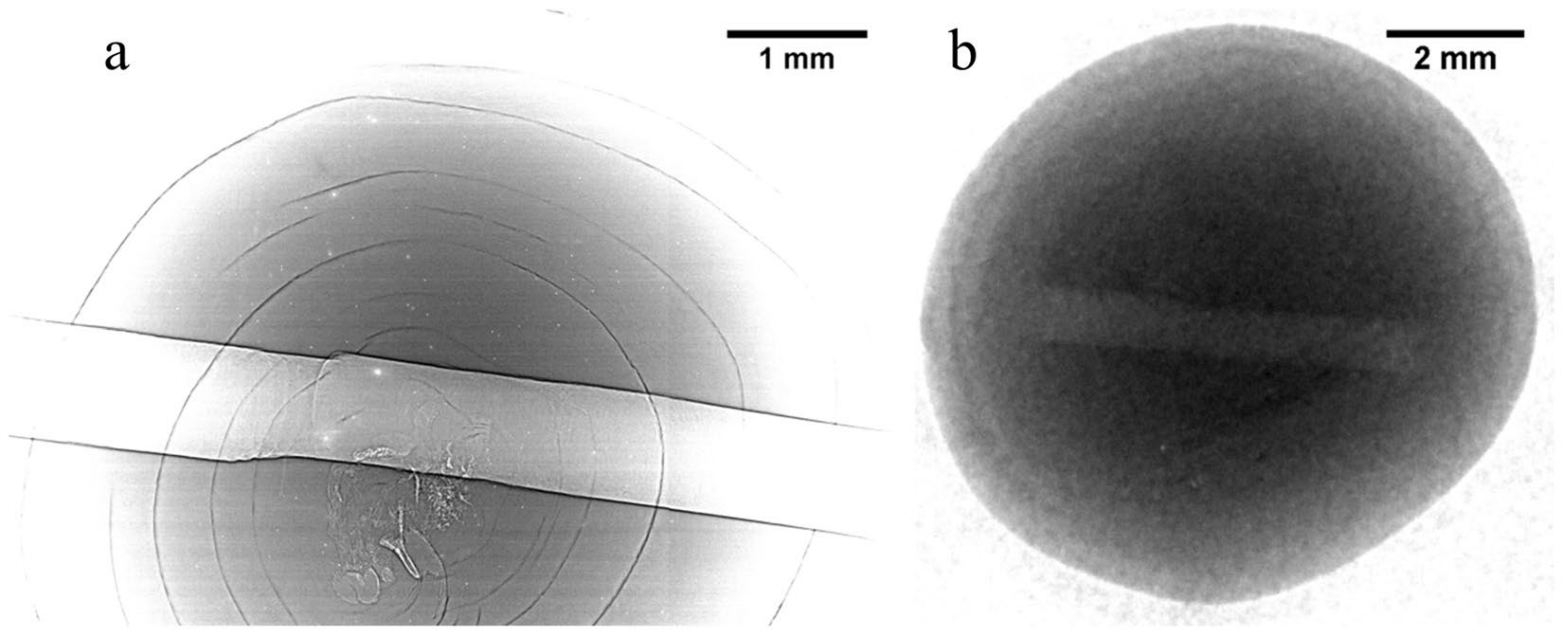
X-ray PC (**a**) and neutron radiographs (**b**) of the cultured freshwater pearl NP-21. In (**a**) the contrast is maximized in order to highlight the growth rings and inner voids of the pearl. The drill-hole is clearly visible in both images.

**Figure 9:**
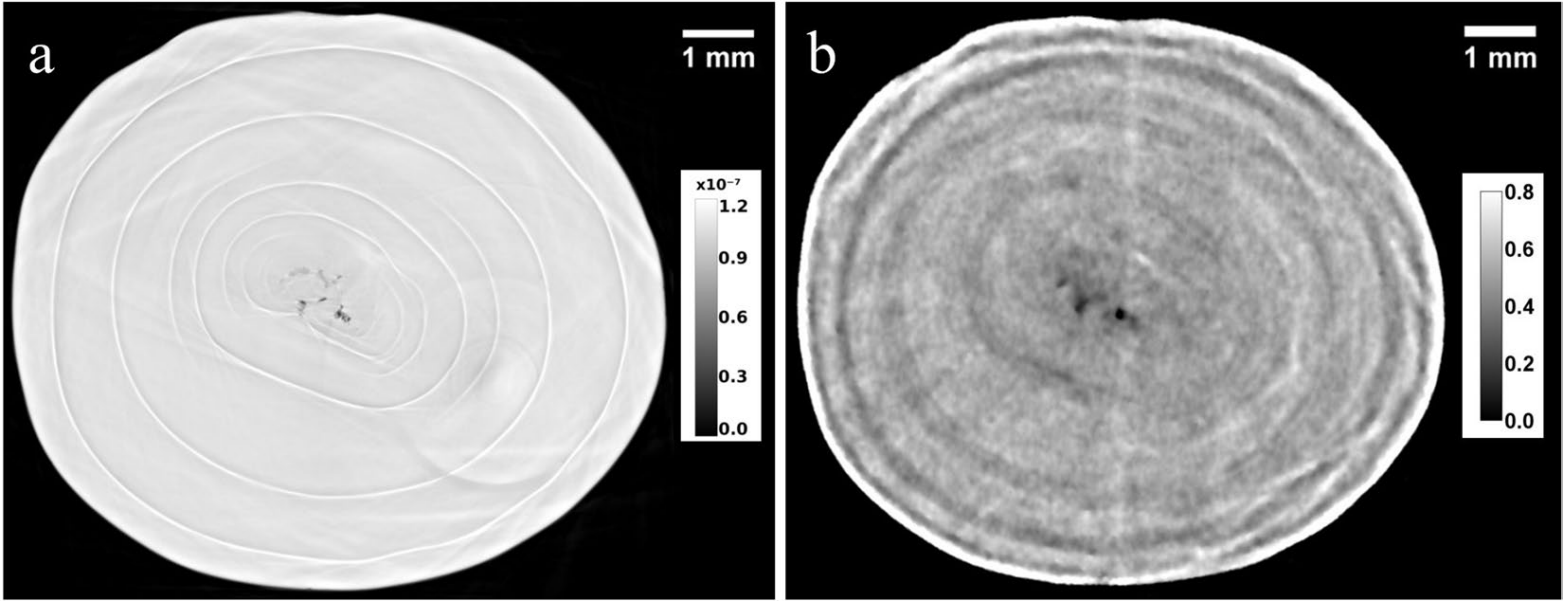
X-ray PC (**a**) and neutron (**b**) CT reconstructed images corresponding to the same slice of the cultured freshwater pearl CP-21. Several growth rings and small voids within the pearl are visible in both reconstructed images. Grey scale refers to refractive index decrement for PCI (**a**) and to linear attenuation coefficient (cm^−1^) for NI (**b**).

**Figure 10:**
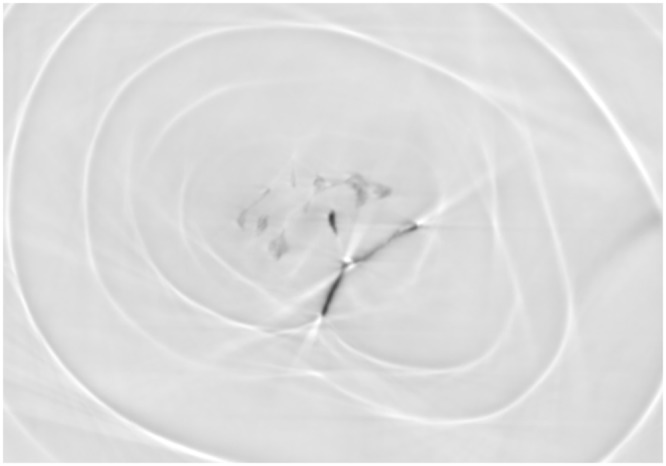
The “moustache” of the cultured freshwater pearl CP-21 revealed by the X-ray PC tomography.

In the case of the pearl CP-21, only the X-ray PCI turned out to be suitable for the identification of the typical structures that characterize the non-bead freshwater pearls. Several growth rings and inner void or cavity-like structures are clearly visible in the X-ray PC radiograph (Fig. 8a), while in a neutron radiograph (Fig. 8b) only a few growth rings barely appear. Both tomographic reconstructions (Fig. 9a,b) reveal the “onion-like” structure of the pearl, but the X-ray PCI allows to visualize in detail some small irregular cavities that in neutron images are scarcely visible^9–11,20^. In particular, the “moustache”, which is the typical fine twisted structural evidence of its origin^18^, can be clearly recognized in the X-ray PC reconstructed slices (Fig. 10) and it proves that the CP-21 is a non-bead cultured pearl.

As a general outlook for applications, we highlight that at PSI it is now possible to perform on-site bimodal imaging with neutrons and X-rays, useful for experiments with low contrast between the relevant features^44^.

Yet, when hydrogen based components are present, further contrast is needed mainly in X-ray imaging, while neutron attenuation will be relevant. Therefore, by using the X-ray PCI technique it is possible to reach higher spatial resolutions, thus allowing a better distinction between organic and inorganic compounds.

In this regard, relevant applications of combined neutron and X-ray imaging have been explored in different fields, like for example materials science, paleontology, medicine. In fact, it has been shown that the two techniques are complementary in studying fossil bones: neutron radiography penetrates hydroxyapatite easier than collagen and, conversely, in X-ray radiography attenuation is higher in hydroxyapatite than in collagen^45^.

In preclinical medical research, it has been shown that metal implants determine artifacts in X-ray images, which determines the ability to accurately quantify the bone healing around the implant. In contrast, neutron images are free of metal artifacts, enabling full analysis of the bone-implant interface^46^.

## Conclusions

In this work, the potentiality of the synchrotron radiation phase-contrast imaging combined with the neutron transmission imaging has been tested as a joint tool to identify complex structures like pearl specimens. Both non-destructive techniques proved to be very promising alternatives to X-ray attenuation-based radiography and micro-tomography in the gemological field, where the identification of pearls as well as the characterization of growing structures may be challenging. In fact, we showed that the major benefit of the adopted techniques with respect to conventional X-ray methods is the capability of discerning small hollow parts and organic materials more clearly. The identification of bead cultured pearls can be achieved reliably by means of either neutron or X-ray PC radiography. However, a radiograph condenses three-dimensional objects into two-dimensional images, so the identification of non-bead cultured pearls and natural ones is in some cases only possible by performing a tomographic scan. We showed that X-ray PC tomography and NI reveal internal features of pearls with great detail - especially X-ray PC technique, due its higher spatial resolution – that allows to make correct pearl identification and help to understand the growing process. Yet, in some cases, neutron images return a better and clearer contrast between organic and inorganic layers, due to the high neutron absorption of H-bearing compounds. This fact results to be helpful for data interpretation in some cases. We finally demonstrated also the potentiality of volume segmentation by using separately neutron and X-ray PC data.

Nevertheless, both tomographic techniques have some limitations. In fact, the reconstructed slices show artifacts, due to the not perfect alignment of the rotation axis respect to the vertical detector axis and to some bad detector pixels that appear in the reconstructed images as small circles and concentric rings, respectively. Although these artifacts can be reduced by pre-processing the projection data, usually they are not completely removed. Hence care must be taken in order to avoid wrong interpretation of these two reconstruction artifacts as onion-like growth structures. Moreover, both tomographic techniques have some disadvantages, unlike the X-ray radiography - the widely used imaging technique by gemologists. In fact, tomographic techniques require longer measurement time, produce large data files, need intensive computational resources for the volume reconstruction and require a trained staff for the data analysis.

Our study revealed biogenetic structures related to the pearls growth, using only non-destructive techniques, combining for the first-time synchrotron-based X-ray PCI and NI reaching a high spatial resolution. The results of this pilot study pave the way to use these combined techniques in the study of the biogenetic mechanisms.

## Data Availability

The datasets generated during and/or analysed during the current study are available from the corresponding author on reasonable request.
